# Self-reported auditory problems are associated with adverse mental health outcomes and alcohol misuse in the UK Armed Forces

**DOI:** 10.1007/s00127-021-02169-8

**Published:** 2021-09-04

**Authors:** Fred N. H. Parker, Nicola T. Fear, S. A. M. Stevelink, L. Rafferty

**Affiliations:** 1 Faculty of Life Sciences and Medicine, Department of Life Sciences and Medicine, King’s College London, London, UK; 2 King’s Centre for Military Health Research, Department of Psychological Medicine, Institute of Psychiatry, Psychology and Neuroscience, King’s College London, London, UK; 3 Academic Department of Military Mental Health, Department of Psychological Medicine, King’s College London, London, UK; 4 Department of Psychological Medicine, Institute of Psychiatry, Psychology and Neuroscience, King’s College London, London, UK

**Keywords:** Armed Forces, Hearing problems, Tinnitus, Mental health, Alcohol misuse

## Abstract

**Purpose:**

Auditory problems, such as hearing loss and tinnitus, have been associated with mental health problems and alcohol misuse in the UK general population and in the US Armed Forces; however, few studies have examined these associations within the UK Armed Forces. The present study examined the association between auditory problems and probable common mental disorders, post-traumatic stress disorder and alcohol misuse.

**Methods:**

5474 serving and ex-service personnel from the UK Armed Forces were examined, selected from those who responded to phase two (data collection 2007–09) and phase three (2014–16) of a military cohort study. Multivariable logistic regression was used to examine the association between auditory problems at phase two and mental health problems at phase three.

**Results:**

9.7% of participants reported ever experiencing hearing problems alone, 7.9% reported tinnitus within the last month alone, and 7.8% reported hearing problems with tinnitus. After adjustment, hearing problems with tinnitus at phase two was associated with increased odds of probable common mental disorders (AOR = 1.50, 95% CI 1.09–2.08), post-traumatic stress disorder (AOR = 2.30, 95% CI 1.41–3.76), and alcohol misuse (AOR = 1.94, 95% CI 1.28–2.96) at phase three. Tinnitus alone was associated with probable post-traumatic stress disorder (AOR = 1.80, 95% CI 1.03–3.15); however, hearing problems alone were not associated with any outcomes of interest.

**Conclusions:**

The association between auditory problems and mental health problems emphasises the importance of the prevention of auditory problems in the Armed Forces: through enhanced audiometric screening, improved hearing protection equipment, and greater levels of utilisation of such equipment.

**Supplementary Information:**

The online version contains supplementary material available at 10.1007/s00127-021-02169-8.

## Introduction

In the UK Armed Forces, auditory problems, the most prevalent being hearing loss and tinnitus, are one of the greatest contributors to disability in personnel [1]. Hearing loss refers to a broad range of deficits to an individual’s hearing, and tinnitus is defined as any noise, although usually ringing, perceived by an individual without an external source. Service in the UK Armed Forces represents a significant risk for auditory problems. Compared to other occupations in the UK, serving members of the Armed Forces suffer the fourth highest level of diagnosed auditory problems [2]. Auditory problems continue to be an issue in those who have left the Armed Forces, with ex-service personnel under 75 years old roughly 3.5 times more likely to report problems with their hearing than the general population [3]. Hearing loss and tinnitus may occur at higher rates in the UK Armed Forces serving and ex-service community than in the general population due to experiences during service, such as exposure to loud noises and head injuries (including injuries causing damage to the auditory system and traumatic brain injury) [4–6]. Illustrating the impact of auditory problems in the UK Armed Forces, the Royal British Legion found that 53% of ex-service personnel with auditory problems reported that their hearing issues had a significant impact on their quality of life; 46% reported that their hearing issues had a significant effect on relationships with friends or family; and 63% reported that their auditory problems had caused them moderate to severe worry, annoyance or upset [3]. Despite this, the majority of personnel may neglect wearing hearing protection regularly on operations, possibly due to issues such as reduced sensory awareness, insufficient training with the hearing protective equipment, or problems with connections between the different built-in communication systems that are used alongside the hearing protective equipment [7]. Without widespread use of hearing protective equipment, auditory problems are likely to continue to be an issue for the UK Armed Forces community.

Auditory problems have been shown to lead to social isolation, emotional distress and issues with employment, all of which are associated with mental health problems [8, 9]. In the general population, self-reported hearing loss was associated with symptoms of anxiety disorders and led to an increased risk of developing depression symptoms later in life, compared to those without hearing loss [10, 11]. Similarly, individuals who rated their tinnitus as severe were found to be at an increased risk of reporting clinically significant anxiety symptoms and depression symptoms, compared to those without tinnitus [12]. Tinnitus may increase levels of anxiety, which, in turn, increases individual’s psychological distress to tinnitus, creating a vicious cycle [13]. Alcohol misuse has been implicated as a maladaptive coping mechanism to tinnitus: individuals who reported that alcohol consumption relieved the symptoms of their tinnitus were found to consume significantly more alcohol than those who reported no effect on their tinnitus [14]. Research within the Armed Forces mirrors the associations between auditory problems and mental health problems seen in the general population. In the US Armed Forces, hearing loss has been significantly associated with depression in older ex-service personnel (mean age 74.3 years) [15]. Diagnoses of tinnitus were associated with diagnoses of anxiety, depression and substance use disorders, including alcohol misuse, in survey of ex-service personnel who receive Department of Veteran’s Affairs health care [16].

Both the prevalence of social issues experienced by UK ex-service personnel with auditory problems, and the evidence of an association between auditory problems and mental health problems from the general population and US military populations, warrants an investigation into the impact of auditory problems on mental health and alcohol misuse among UK serving and ex-service personnel. However, there is a lack of studies exploring the impact of hearing loss and tinnitus on mental health in the UK Armed Forces over time. The present study has two primary aims using data from UK serving and ex-service Armed Forces personnel, namely to:

1. describe the prevalence of auditory problems, and
2. examine the long-term effects of auditory problems on mental health, considering a range of associated sociodemographic and military service factors

It is hypothesised that serving and ex-service personnel who report auditory problems (hearing problems and/or tinnitus) will be more likely to meet the caseness criteria for probable common mental disorders (CMD), post-traumatic stress disorder (PTSD) and alcohol misuse, compared to those without auditory problems.

## Methods

### Study design and participants

Since 2003, the King’s Centre for Military Health Research (KCMHR) has been conducting a longitudinal observational study on the health and wellbeing of the UK Armed Forces, including both serving and ex-service personnel covering three phases of data collection to date. The initial phase of data collection (2004–06) sought to examine the effect of deployment on operations to Iraq (codename operation TELIC) on the physical and mental health of UK Armed Forces personnel [17]. The phase one sample included personnel who were deployed to Iraq in 2003 described as the TELIC group. The comparison group (termed Era) were personnel who were not deployed during that time, despite being eligible for deployment (*n* = 10,272, response rate 58.0%) [17]. A subsequent phase of data collection was conducted between 2007 and 2009. The phase two sample included participants from phase one who consented to further contact, a sample of personnel deployed to Afghanistan (codename operation HERRICK) between 2006 and 2007, and a replenishment group of regular and reserve personnel who had joined the Armed Forces between 2003 and 2007 (*n* = 10,290, response rate 58.8%) [18]. Between 2014 and 2016, data was collected for phase three, including participants who consented to further contact in phase two, termed the follow up sample, and another replenishment sample (*n* = 8093, response rate 44.3%) [19]. The results presented within this paper report on serving and ex-service personnel with complete data who responded to both phase two and phase three (*n* = 5474).

### Materials

The KCMHR health and well-being cohort study collects self-report data on sociodemographic background, military service history, experiences during deployment, health (physical and mental), lifestyle behaviours and relationships. Participants received self-complete questionnaires through post, base visits or via an email link (for phase three only). At phase two, participants were asked if they had ever experienced hearing problems (Yes/No), and if they had experienced ringing in their ears in the past month (Yes/No), which was used as an indication of tinnitus, similar to other literature [1, 3, 20]. Responses to the auditory problems questions were combined to make a single “auditory problems at phase two” variable with four categories: no auditory problems, hearing problems alone, tinnitus alone, and hearing problems with tinnitus. Mental health was assessed using the 12-item General Health Questionnaire (GHQ-12), the 17-item National Centre for PTSD Civilian Checklist (PCL-C), and the 10-item World Health Organisation’s Alcohol Use Disorders Identification Test (AUDIT). Binary outcome variables were defined using the following cut-off scores: 4 or more on the GHQ-12 (score range 0–12) indicated probable CMD [21], 50 or more on the PCL-C (score range 17–85) indicated probable PTSD [22], and 16 or more on the AUDIT (score range 0–40) indicated alcohol misuse [23]. The cut-offs described above have all been widely used and validated in Armed Forces populations [24–26].

### Statistical analysis

Both samples (phase two and phase three) were combined into a single dataset. Sociodemographic, health and military service factors were described using the full sample of the cohort at phase two. The associations between auditory problems and sociodemographic or military service factors at phase two were examined. Multivariable logistic regression was performed to examine whether auditory problems at phase two were associated with probable CMD, probable PTSD or alcohol misuse at phase three, using no auditory problems as the referent category. Multivariable logistic regressions were conducted between auditory problems at phase two and outcomes of interest as described above, thereby adjusting for variables that may affect the associations. Adjustments were decided upon a priori based on previous literature, grouped into sociodemographic factors, military service factors and probable mental health problems at phase two [18, 19, 27]. A stepwise approach was followed, whereby variables derived at phase two were adjusted for in sequence;

1. Sociodemographic factors (gender, age, relationship status and education level).
2. Sociodemographic and military service factors (rank, service branch, regular/reservist, role in parent unit and deployment to Iraq or Afghanistan).
3. Sociodemographic, military service factors and past mental health problems (probable CMD, probable PTSD) and alcohol misuse at phase two.

All statistical analyses were performed using the statistical package Stata, version 15.0, with survey commands used to account for weighting. Sample weights were calculated as the inverse probability of being sampled from a specific subpopulation (Era, TELIC, HERRICK, phase two replenishment). Response weights were generated to account for non-responders calculated as the inverse probability of responding once sampled. Sample weights were multiplied by response weights to give a single probability weight. Weighted percentages, odds ratios (ORs) and adjusted odds ratio (AOR) with 95% confidence intervals (95% CIs) are presented in the tables alongside unweighted cell counts. Missing data were dropped from the main analysis. In general, less that 2.5% of responses to the outcome or exposures of interest contained missing data, however hearing problems had a higher percentage (10.4%). Sensitivity analysis was run. Individuals who had missing data on hearing problems at phase two were more likely to report probable PTSD (OR = 2.15, 95% CI 1.45–3.18) and alcohol misuse (OR = 1.43, 95% CI 1.01–2.02) at phase three. Conversely, missing data on tinnitus was not associated with any of the outcomes of interest (data available on request).

### Ethical approval

The results reported in this paper are from secondary analysis of existing data which falls under the original ethical approval granted by the UK Ministry of Defence Research Ethics Committee and the King’s College London Psychiatry, Nursing, and Midwifery Research Ethics Subcommittee. All participants provided written informed consent for their data being used for research purposes and the subsequent publication of the findings.

## Results

Of the 5474 serving and ex-serving personnel included in the analysis, the mean age at phase two was 37.1 years (range 18–66). The majority of the participants were male (89.0%), still serving (70.9%) and regular serving personnel (87.5%), as opposed to reservist personnel. Over sixty percent of participants were in the Army (61.3%), with 21.7% in the Royal Air Force (RAF) and 17.0% in the Naval Services.

### Prevalence of auditory problems

One-thousand two-hundred and five participants reported some degree of auditory problems at phase two. Hearing problems alone were reported by 9.7% (95% CI 8.7–10.7) of participants; tinnitus alone was reported by 7.9% (95% CI 7.0–8.8) of participants; and hearing problems with tinnitus were reported by 7.8% (95% CI 7.0–8.7) of participants (Table 1). The majority of individuals who reported hearing problems or tinnitus at phase two reported that they were still bothered by hearing problems or tinnitus at phase three, illustrating the longevity of auditory problems (supplementary table 1). In general, older participants and males were more likely to report auditory problems than younger participants and females. Individuals who had left the Armed Forces or served in a combat role (compared to combat support and combat service support) reported higher proportions of all auditory problems. Comparing the branches of the Armed Forces (Naval Services, Army, and RAF), individuals in the Naval Services reported higher proportions of hearing problems alone whereas individuals in the Army reported higher proportions of tinnitus alone and hearing problems with tinnitus. Non-Commissioned Officers (NCOs) reported higher proportions of hearing problems and hearing problems with tinnitus compared to officer or other ranks, whereas those of other ranks reported higher proportions of tinnitus alone. Finally, individuals who had not been deployed reported higher proportions of hearing problems alone compared to those who had deployed.

**Table 1:** Description of study participants (sociodemographic and military service factors) by auditory problems at phase 2 (total *n* = 5474)

		Auditory problems at phase two				
Phase 2 variables	Number of participants	No auditory problems	Hearing problems alone	Tinnitus alone	Hearing problems with tinnitus	*p*^a^
	*n*	*n* (%)^b^	*n* (%)^b^	*n* (%)^b^	*n* (%)^b^	
Total	5474	3717 (74.7)	437 (9.7)	391 (7.9)	377 (7.8)	
Age group (in years)						
< 25	502	382 (80.0)	29 (6.7)	45 (8.7)	21 (4.5)	
25–29	948	743 (82.9)	36 (4.8)	62 (6.7)	46 (5.7)	
30–34	927	682 (81.3)	52 (5.8)	62 (7.8)	46 (5.2)	
35–39	1048	703 (73.8)	81 (9.6)	89 (9.3)	68 (7.3)	
40–50	1557	968 (70.9)	169 (12.8)	98 (7.3)	128 (9.0)	
> 50	492	239 (55.1)	70 (19.3)	35 (8.4)	68 (17.2)	** < 0.001**
Gender						
Male	4787	3178 (73.1)	408 (10.3)	362 (8.3)	357 (8.3)	
Female	687	539 (87.4)	29 (4.5)	29 (4.7)	20 (3.4)	** < 0.001**
Relationship status						
In a relationship	4309	2877 (73.6)	362 (10.1)	303 (7.9)	322 (8.4)	
Not in a relationship	1145	826 (79.4)	74 (7.6)	87 (8.0)	53 (4.9)	**0.002**
Education level						
Low^c^	1881	1216 (72.2)	162 (10.5)	149 (8.5)	144 (8.7)	
High^d^	3353	2355 (76.7)	245 (8.7)	227 (7.6)	214 (7.1)	**0.036**
Self-reported health						
Excellent / very good	3223	2438 (80.3)	207 (7.5)	208 (7.2)	160 (5.0)	
Good	1644	999 (69.9)	167 (12.5)	125 (7.9)	134 (9.8)	
Fair / poor	591	279 (56.8)	63 (13.9)	56 (11.7)	82 (17.6)	** < 0.001**
Smoking status						
No	4432	3058 (75.7)	353 (9.5)	314 (7.8)	277 (7.0)	
Yes	1017	646 (70.1)	83 (10.5)	77 (8.2)	99 (11.2)	**0.002**
Service branch						
Naval services	841	553 (75.3)	75 (11.4)	48 (6.1)	54 (7.2)	
Army	3450	2319 (73.1)	259 (9.2)	284 (9.1)	259 (8.6)	
RAF	1183	845 (78.6)	103 (9.7)	59 (5.9)	64 (5.9)	**0.002**
Engagement type						
Regular	4359	2970 (74.6)	360 (9.9)	309 (7.9)	294 (7.5)	
Reservist	1115	747 (74.9)	77 (8.1)	82 (7.4)	83 (9.6)	0.172
Rank						
Officer	1594	1146 (77.9)	117 (8.5)	95 (7.0)	97 (6.6)	
NCO	3083	1991 (72.4)	283 (11.1)	222 (7.8)	243 (8.8)	
Other	797	580 (79.4)	37 (5.2)	74 (10.2)	37 (5.2)	** < 0.001**
Previous deployment						
No	2964	1887 (73.0)	261 (11.3)	187 (7.4)	205 (8.4)	
Yes^e^	2510	1830 (76.7)	176 (7.8)	204 (8.4)	172 (7.1)	**0.002**
Serving status						
Serving	4043	2896 (77.3)	304 (8.7)	285 (7.5)	250 (6.5)	
Discharged	1420	815 (67.5)	132 (12.4)	105 (8.8)	127 (11.3)	** < 0.001**
Role in parent unit						
Combat	1115	685 (66.1)	107 (10.9)	114 (11.5)	112 (11.5)	
Combat support	646	460 (77.6)	48 (8.7)	55 (9.4)	23 (4.4)	
Combat service support	3671	2541 (76.7)	279 (9.5)	219 (6.5)	241 (7.3)	** < 0.001**

Bold indicates statistical significance (*p* < 0.05)

*RAF* Royal Air Force. Naval Services, combines the Royal Navy and Royal marines, *NCO* non-commissioned officer, *GCSE* general certificate of secondary education

^a^*p* value via Pearson’s Chi-squared test.

^b^Numbers are unweighted, percentages are weighted

^c^O levels, GCSE or none

^d^A level, degree or higher

^e^Deployed to Iraq and/or Afghanistan

### Associations between auditory problems and mental health problems

Generally, the prevalence of mental health problems and alcohol misuse at phase three was higher in individuals who reported any auditory problems at phase two compared to those who reported no auditory problems. However, individuals with hearing problems alone reported lower proportions of alcohol misuse compared to those with no auditory problems (Table 2).

**Table 2:** Prevalence of probable mental health problems and alcohol misuse at phase three by auditory problems at phase two (total *n* = 5474)

	Number of participants	CMD		PTSD		Alcohol misuse	
	*n*	*n* (%)^a^	*p*^b^	*n* (%)^a^	*p*^b^	*n* (%)^a^	*p*^b^
Total	5474	996 (20.0)		232 (4.7)		428 (8.7)	
No auditory problems	3717	685 (18.3)		135 (3.7)		305 (8.2)	
Hearing problems alone	437	93 (21.7)		19 (3.9)		29 (6.3)	
Tinnitus alone	391	102 (24.3)		34 (8.8)		37 (8.8)	
Hearing problems with tinnitus	377	116 (29.3)	** < 0.001**	44 (11.2)	** < 0.001**	57 (16.3)	** < 0.001**

Bold indicates statistical significance (*p* < 0.05)

*CMD* common mental disorders, *PTSD* post-traumatic stress disorder

^a^Numbers are unweighted, percentages are weighted

^b^*p* value via Pearson’s Chi-squared test.

The associations between auditory problems and mental health outcomes and alcohol misuse were examined (Tables 3, 4, and 5). After full adjustment, hearing problems with tinnitus reported at phase two was found to significantly increase the odds of probable CMD at phase three (AOR = 1.50, 95% CI 1.09–2.08). Additionally, there was borderline significance between hearing problems alone and tinnitus alone at phase two and probable CMD at phase three (AOR = 1.35, 95% CI 0.99–1.85 and AOR = 1.35, 95% CI 0.99–1.84, respectively). The odds of reporting probable PTSD at phase three was significantly increased in those who reported tinnitus alone (AOR = 1.80, 95% CI 1.03–3.15) and hearing problems with tinnitus (AOR = 2.30, 95% CI 1.41–3.76) at phase two. Finally, hearing problems with tinnitus reported at phase two significantly increased the odds of alcohol misuse at phase three (AOR = 1.94, 95% CI 1.28–2.96) after the full adjustment, whereas reports of hearing problems alone or tinnitus alone were not associated with alcohol misuse. To summarise, in the fully adjusted model, individuals who reported hearing problems with tinnitus at phase two had roughly 1.5 times the odds of reporting probable CMD, 2.5 times the odds of probable PTSD, and twice the odds of alcohol misuse at phase three.

**Table 3:** Association between auditory problems at phase two and probable CMD at phase three

	Number of Participants	Probable CMD at phase three				
	*n* (%)^a^	*n* (%)^a^	OR (95% CI)	AOR1^b^ (95% CI)	AOR2^c^ (95% CI)	AOR3^d^ (95% CI)
No auditory problems	3717 (74.7)	685 (68.4)	1	1	1	1
Hearing problems alone	437 (9.7)	93 (10.4)	1.24 (0.93–1.65)	**1.42 (1.05–1.93)**	**1.44 (1.06–1.95)**	1.35 (0.99–1.85)
Tinnitus alone	391 (7.9)	102 (9.7)	**1.43 (1.07–1.91)**	**1.50 (1.11–2.02)**	**1.48 (1.10–1.98)**	1.35 (0.99–1.84)
Hearing problems with tinnitus	377 (7.8)	116 (11.5)	**1.84 (1.40–2.43)**	**2.03 (1.52–2.72)**	**1.97 (1.47–2.64)**	**1.50 (1.09–2.08)**

Bold indicates statistical significance.

*CMD* common mental disorders, *OR* odds ratio, *AOR* adjusted odds ratio.

^a^Numbers are unweighted, percentages are weighted

^b^AOR1—adjusted for sociodemographic factors (gender, age (continuous), relationship status, education level)

^c^AOR2—adjusted for the sociodemographic factors and military service factors (rank, service branch, regular/reservist, role in parent unit and deployment to Iraq or Afghanistan)

^d^AOR3—adjusted for sociodemographic factors, military service factors and mental health problems (probable CMD, probable PTSD) and alcohol misuse at phase two

**Table 4:** Association between auditory problems at phase two and probable PTSD at phase three

	Number of Participants	Probable PTSD at phase three				
	*n* (%)^a^	*n* (%)^a^	OR (95% CI)	AOR1^b^ (95% CI)	AOR2^c^ (95% CI)	AOR3^d^ (95% CI)
No auditory problems	3717 (74.7)	135 (58.5)	1	1	1	1
Hearing problems alone	437 (9.7)	19 (8.0)	1.06 (0.59–1.92)	1.37 (0.74–2.54)	1.30 (0.70–2.41)	0.96 (0.53–1.74)
Tinnitus alone	391 (7.9)	34 (15.0)	**2.53 (1.56–4.11)**	**2.73 (1.66–4.49)**	**2.19 (1.35–3.56)**	**1.80 (1.03–3.15)**
Hearing problems with tinnitus	377 (7.8)	44 (18.5)	**3.29 (2.15–5.04)**	**4.13 (2.62–6.51)**	**3.40 (2.13–5.43)**	**2.30 (1.41–3.76)**

Bold indicates statistical significance.

*PTSD* post-traumatic stress disorder, *OR* odds ratio, *AOR* adjusted odds ratio.

^a^Numbers are unweighted, percentages are weighted

^b^AOR1—adjusted for sociodemographic factors (gender, age (continuous), relationship status, education level)

^c^AOR2—adjusted for the sociodemographic factors and military service factors (rank, service branch, regular/reservist, role in parent unit and deployment to Iraq or Afghanistan)

^d^AOR3—adjusted for sociodemographic factors, military service factors and mental health problems (probable CMD, probable PTSD) and alcohol misuse at phase two

**Table 5:** Association between auditory problems at phase two and alcohol misuse at phase three

	Number of Participants	Alcohol misuse at phase three				
	*n* (%)^a^	*n* (%)^a^	OR (95% CI)	AOR1^b^ (95% CI)	AOR2^c^ (95% CI)	AOR3^d^ (95% CI)
No auditory problems	3717 (74.7)	305 (70.2)	1	1	1	1
Hearing problems alone	437 (9.7)	29 (7.0)	0.76 (0.47–1.23)	0.77 (0.46–1.28)	0.77 (0.47–1.29)	0.72 (0.44–1.17)
Tinnitus alone	391 (7.9)	37 (8.1)	1.08 (0.70–1.66)	1.08 (0.69–1.68)	1.06 (0.68–1.66)	0.92 (0.54–1.56)
Hearing problems with tinnitus	377 (7.8)	57 (14.7)	**2.19 (1.53–3.14)**	**2.46 (1.70–3.58)**	**2.38 (1.64–3.46)**	**1.94 (1.28–2.96)**

Bold indicates statistical significance.

*OR* odds ratio, *AOR* adjusted odds ratio.

^a^Numbers are unweighted, percentages are weighted

^b^AOR1—adjusted for sociodemographic factors (gender, age (continuous), relationship status, education level)

^c^AOR2—adjusted for the sociodemographic factors and military service factors (rank, service branch, regular/reservist, role in parent unit and deployment to Iraq or Afghanistan)

^d^AOR3—adjusted for sociodemographic factors, military service factors and mental health problems (probable CMD, probable PTSD) and alcohol misuse at phase two

## Discussion

This paper examined the associations between past auditory problems (specifically self-reported hearing problems and self-reported tinnitus), and current mental health problems (specifically probable CMD and PTSD) or alcohol misuse using data from serving and ex-service UK Armed Forces personnel at two timepoints. Our findings are three-fold:

1. Hearing problems alone mildly increased the odds of reporting probable CMD, although no significant associations were found between hearing problems alone and any outcomes of interest.
2. Those who reported tinnitus alone were more likely to report probable PTSD, although not CMD or alcohol misuse, and
3. Those who reported hearing problems with tinnitus were indeed more likely to report all three outcomes of interest (probable CMD, probable PTSD, and alcohol misuse).

### Prevalence of auditory problems

The present proportions of self-reported hearing loss and tinnitus among UK Armed Forces serving and ex-service personnel were more prevalent than those found in studies of the general UK population, adding support to the assertion that military service may represent a risk factor for auditory problems [28–30]. Direct comparisons between the UK general population and the present sample are difficult due to variations of the indicators of hearing problems and tinnitus, and the importance of matching the sociodemographic factors (such as age and gender) of the sample. Taking this into account, the prevalence of auditory problems was considerably higher in the present study compared to the Royal British Legion – Survey of the Ex-service Community, with similar questions used to indicate hearing problems and tinnitus in both studies [3]. It is possible that an increased use of improvised explosive devices in recent conflicts may have led to a higher prevalence of auditory problems in the present sample due to blast injuries and greater noise exposure [31, 32]. A systematic review of studies relating to auditory problems in US Armed Forces after deployment to Iraq or Afghanistan, suggested that representative estimates of self-reported hearing problems across the whole Armed Forces range from 7.3% to 26.6% [33]. The present findings fall within this range, suggesting a similarity in auditory problems within the UK and US Armed Forces.

Auditory protection and screening are important in the prevention of auditory problems. The most modern hearing protection equipment in the UK Armed forces is the Personnel Interfaced Hearing Protection (PIHP) system, which replaced the Combat Arms Ear Plug in 2009. The PIHP is an electronic system that relays loud noises to the wearer’s ears at a safe level, providing hearing protection with little sensory inattention, and has the ability to couple with the user’s Personal Role Radio. The PIHP system was introduced at the end of phase two data collection (2007–09), so the benefits of the newer system may not be observed in our sample. Alternatively, a low compliance may have reduced the effectiveness of the PIHP system. As such, despite the improvements in auditory personal protective equipment, a survey of a Forward Operating Base in Afghanistan found that only 4% of individuals routinely wore PIPH on patrol due to fears of sensory inattention, communication issues and inadequate training [7]. In the UK Armed Forces, routine audiometric testing is performed on recruitment and discharge under the PULHHEEMS (Physical capacity, Upper limbs, Locomotion, Hearing (right and left), Eyesight (right and left), Mental function, Stability (emotional)) assessment, as well as yearly and 6 months before and after deployment, as part of the UK Military Hearing Conservation Programme [34]. Individuals who are suspected to have auditory problems may receive improved auditory personal protective equipment, modified service away from areas of high noise, or medical discharge. However, blast injuries or mild-traumatic brain injury (mTBI) may lead to auditory problems through damage to the underlying neuronal structure without damaging the peripheral hearing system, such as the cochlea. In these individuals, the presence of a normal audiometric test does not exclude damage to auditory system which may exist in the central auditory system, such as the auditory cortex [35]. Moreover, tinnitus is not routinely examined specifically and some individuals with tinnitus may have normal audiometric testing. If auditory problems are not detected on audiometric screening, individuals may not receive appropriate additional supportive measures before their auditory problems worsen and significantly impact their quality of life.

In the present study, the associations found between auditory problems and sociodemographic factors align with literature from the general population [8, 32]. Individuals who had left the Armed Forces were more likely to report all auditory problems compared to those still serving. Ex-service personnel tend to be older than personnel still serving, with higher prevalence of hearing problems observed in the older aged groups, which may explain this relationship. However, it is possible that individuals with auditory problems were more likely to be discharged for medical reasons or elect to leave the Armed Forces. Alternatively, individuals might be more willing to disclose auditory problems once they have left the Armed Forces due to fears that reporting auditory problems whilst still serving may have impacted their military careers (especially impacting their fitness for deployment). It is possible that different military service experiences may lead to different amounts of damaging noise exposure and variable auditory problems [36]. In the US Armed Forces, individuals who experienced combat while deployed were more likely to report hearing loss compared to those who were deployed but did not experience combat [31]. This was believed to be due to increased exposure to damaging noise, such as gunfire or explosions, and reduced compliance with auditory protective equipment. The present study also found that those in combat roles reported higher proportions of auditory problems than those in support roles. In a study of US personnel who were deployed to Iraq or Afghanistan, lower rank was associated with increased risk of hearing loss similar to the current findings [32]. In the present study, higher proportions of hearing problems were reported in those with no previous deployments whereas higher proportions of tinnitus alone were reported in those who had been deployed. It may be that those who experienced hearing problems were less likely to be deployed due to poor health, were more likely to be discharged for medical reasons or elect to leave the Armed Forces before they were deployed [37]. Tinnitus may not affect deployment to the same extent and may occur at higher proportions in those who were deployed due to combat exposures while deployed such as gunfire, blast injuries and mTBI [38].

### Associations between auditory problems and mental health problems and alcohol misuse

After full adjustment, hearing problems with tinnitus was associated with an increased odds of probable CMD. Auditory problems may lead to isolation and social withdrawal due to resultant communication issues [8]. Moreover, tinnitus can result in discomfort when exposed to high levels of noise which may affect an individuals’ desire to attend social interactions that occur in loud areas, such as bars and restaurants, exacerbating social isolation [39]. These communication issues and impacts on social interactions could all affect an individual’s social network, which have been associated with CMD, chiefly depression and anxiety disorders, which may be the case in the present sample [40, 41].

Tinnitus was associated with probable PTSD, both when experienced alone and when combined with hearing problems. The odds of probable PTSD was similar comparing tinnitus alone and hearing problems with tinnitus, suggesting that tinnitus may be the main driver of this relationship. While the associations could also be explained by the symptom overlap between tinnitus and PTSD, as the associations remained after adjustment for probable PTSD at phase two, auditory problems and probable PTSD are unlikely to be simply co-occurring. In the Armed Forces, a temporary tinnitus may occur due to the noise emitted by gunfire or explosions during traumatic events that may precipitate PTSD. By occurring simultaneously, tinnitus may become associated with traumatic events through classical conditioning [42]. If tinnitus persists or re-occurs later in life, it may act as reminder of traumatic events, potentiating the sense of re-experiencing in PTSD. Moreover, tinnitus may trigger the recall of traumatic memories, which may increase their persistence and intrusiveness, leading to the development or worsening of PTSD symptoms [43]. Alternatively, tinnitus and PTSD may be caused by the same event. Previous research indicated that sustaining mTBI was associated with an increased risk of reporting both ringing in the ears and symptoms of PTSD [38]. In the present sample, hearing problems alone was not associated with probable PTSD. It may be that because of the treatable nature of hearing problems, as opposed to the difficulties associated with treating tinnitus, hearing problems are less of a persistent reminder of traumatic events [44]. In contrast to the present findings, a recent study in the US Armed Forces found that hearing loss was associated with a greater burden of PTSD [45]. However, the US study only examined people who sustained hearing loss through injuries during the Iraq or Afghanistan conflicts, suggesting that the cause of auditory problems may affect these associations. These findings suggest that combat exposures may have an important role in the relationship between auditory problems and PTSD, and an important future research direction.

Those who reported hearing problems with tinnitus were found to be at an increased odds of reporting alcohol misuse later in life. Multiple pathways may explain the association between auditory problems and alcohol misuse. Auditory problems may lead to emotional distress, social isolation and issues with employment, all of which may increase risk of alcohol misuse [3, 46]. Alternatively, individuals may misuse alcohol as a maladaptive coping mechanism to the psychological distress that may be caused by auditory problems. Indeed some individuals report that alcohol can reduce the symptoms of tinnitus that they experience: a study on the general public of the UK found that individuals who reported that alcohol had beneficial effects on their tinnitus consumed significantly more units of alcohol per week compared to those who reported no benefit [14]. Additionally, alcohol misuse is often seen in conjunction with mental health issues, as seen in the present study.

### Implications

Auditory problems were reported at a substantial level across this sample of UK Armed Forces serving and ex-service personnel. For future research, it may be helpful to validate the number of auditory problems found by comparing them with findings of the PULHHEEMS or Hearing Conservation Programme, as self-reported auditory problems may be reported inaccurately depending on factors such as age, occupational noise exposure or diagnoses of mental disorders [47]. A survey of the levels of noise exposure across branches in the UK Armed Forces may help to ensure that individuals are supplied with an adequate level of hearing protection equipment to help prevent auditory problems. Such surveys have already been performed by the Royal Navy under the Hearing Conservation Programme, in accordance with The Control of Noise at Work Regulations, 2005 [48]. In the Armed Forces, low compliance is often cited as a barrier to effective auditory system protection, as is incorrect use of equipment. Therefore, individuals in the UK Armed Forces may benefit from additional teaching in how to correctly use their hearing protection equipment, especially during training and practice exercises, and increased awareness of auditory problems may improve compliance. Individuals in the Armed Forces may benefit from improved audiometric screening so that greater auditory protective measures can be implemented before the auditory problems develop further and affect quality of life. Addressing these issues, the Remote Audiometric Performance Innovation Evaluation and Review (RAPIER) project is a recent directive aimed to improve the detection, referral and management of those with auditory problems.

Self-reported auditory problems were shown to be associated with later reports of probable mental health problems and alcohol misuse. This emphasises the importance to recognise and treat auditory problems as early as possible, before mental health problems develop. Self-reported auditory problems may occur in individuals without a biological cause as a medically unexplained symptom through somatisation of psychological distress [49]. Thus, it may be helpful to view self-reported auditory problems as non-specific indicators of psychological distress and pre-cursors to mental health issues and alcohol misuse. Moreover, mental health services may wish to identify individuals with auditory problems as of potentially higher risk for developing mental health problems, providing additional support to this group. For example, individuals with auditory problems may benefit from communication support to help reduce social isolation. Additionally, developing evidence-based treatment protocols for those who are suffering from co-morbid auditory problems and mental health problems may better address their specific treatment needs, such as Eye Movement Desensitisation and Reprocessing therapy to treat both tinnitus and PTSD concurrently [50, 51].

### Strengths and limitations

Some limitations of the study are important to note. The sensitivity analysis found that individuals with missing data on hearing problems were more likely to report probable PTSD and alcohol misuse. This may have led to an underestimation of the association between hearing problems alone and probable PTSD or alcohol misuse. The questionnaire relied on self-report of auditory problems, which may be over-reported due to negative affect [47]. However, the adjustment for mental health problems at phase two makes it unlikely that this would invalidate conclusions. Hearing problems were defined as having ever experienced a problem with hearing, therefore individuals may have reported previous hearing problems that have resolved. Moreover, the auditory problems that some individuals reported at phase two may have resolved when mental health problems and alcohol misuse were screened at phase three. This suggests that another factor may have contributed to the outcomes of the present study. Tinnitus was defined as experiencing ringing in the ears over the past month, similar to other studies in UK Armed Forces, however pathological status may be argued to require occurrence over a year [52]. While appropriate audiometric testing would increase the accuracy of hearing loss screening, tinnitus lacks clear diagnostic criteria, and audiometric testing would not be feasible on a sample of this size and scope. It is difficult to ascertain if auditory problems and mental health problems were related to the military service, or due to events before or after leaving service, with no way to prove military service as the cause of either. Key strengths of the current study include its large sample size and detailed information collected on sociodemographic, military service and mental health factors.

## Conclusion

In the present sample of the UK serving and ex-service Armed Forces, around one in four individuals reported some degree of auditory problems. After the full adjustment, past reports of tinnitus alone were associated with current probable PTSD and hearing problems with tinnitus were associated with probable CMD, PTSD, and alcohol misuse. Members of the UK Armed Forces may benefit from a review of auditory protective equipment, ensuring that individuals have sufficient training with appropriate equipment for the levels of noise exposure, increase compliance and improved methods of auditory screening.

## Supplementary Information

Below is the link to the electronic supplementary material.

Supplementary file1 (DOCX 22 KB)

## Data Availability

Data supplied as part of the King’s Centre for Military Research Health and Well-being cohort study.
