# Supplementary material for: Self-reported auditory problems are associated with adverse mental health outcomes and alcohol misuse in the UK Armed Forces
Source: Soc Psychiatry Psychiatr Epidemiol. 2021 Sep 4;57(3):563–73. doi: 10.1007/s00127-021-02169-8 (PMC8934320; doi:10.1007/s00127-021-02169-8)
Supplement: Supplementary file 1 — Supplementary file1 (DOCX 22 KB) [file 127_2021_2169_MOESM1_ESM.docx]

# **Supplementary tables**

**Supplementary table 1**

| Supplementary table 1: Impact of auditory problems at phase 3 by auditory problems at phase 2 (total n = 5474) | | | | | | |
| --- | --- | --- | --- | --- | --- | --- |
|  |  | **Auditory problems at phase two** | | | | |
| Auditory problems at phase three | **Number of participants** | **No auditory problems** | **Hearing problems alone** | **Tinnitus alone** | **Hearing problems with tinnitus** | **p^b^** |
|  | n | n (%)^a^ | n (%)^a^ | n (%)^a^ | n (%)^a^ |  |
| Total | 5474 | 3717 (74.7) | 437 (9.7) | 391 (7.9) | 377 (7.8) |  |
| Hearing problems |  |  |  |  |  |  |
| Not bothered at all | 3606 | 3064 (84.8) | 195 (5.7) | 254 (7.0) | 93 (2.6) |  |
| Bothered a little | 888 | 476 (51.9) | 180 (11.9) | 103 (11.9) | 129 (13.9) |  |
| Bothered a lot | 340 | 106 (28.2) | 58 (18.0) | 28 (7.8) | 148 (46.0) | **<0.001** |
| Tinnitus |  |  |  |  |  |  |
| Not bothered at all | 3448 | 2936 (84.7) | 300 (9.2) | 137 (3.8) | 75 (2.3) |  |
| Bothered a little | 924 | 527 (55.2) | 97 (12.3) | 164 (17.8) | 136 (14.7) |  |
| Bothered a lot | 159 | 176 (39.3) | 33 (7.3) | 85 (18.9) | 159 (34.5) | **<0.001** |
| 1. Numbers are unweighted, percentages are weighted. 2. P value via Pearson’s Chi-squared test | | | | | | |
